# Desmoplastic (collagenous) fibroma of the femur: A case report and review of the literature

**DOI:** 10.3892/ol.2013.1535

**Published:** 2013-08-19

**Authors:** SONGTAO GAO, QIQING CAI, WEITAO YAO, JIAQIANG WANG, PENG ZHANG, XIN WANG

**Affiliations:** Department of Orthopedics of Henan Tumor Hospital, The Affiliated Tumor Hospital of Zhengzhou University, Zhengzhou, Henan 450008, P.R. China

**Keywords:** desmoplastic fibroma, collagenous fibroma, femur, thigh mass, fibroblastic, internal fixation

## Abstract

Desmoplastic fibroma is a rare, benign soft-tissue tumor composed of spindled and stellate-shaped cells that are embedded in a dense collagenous stroma. Clinically, desmoplastic fibroma presents as a firm, mobile, slow-growing mass that is located in the subcutaneous tissue or near the deep aspect of the skeletal muscles. The present study describes the case of a 66-year-old female who presented with an inactive, firm, slightly tender mass in the lower medial segment of the right femur. An open biopsy was performed and the result of the pathological examination indicated a desmoplastic fibroma. The patient underwent a radical resection of the tumor and the accompanying bone, which was then reimplanted using devitalized tumor bone, self-ilium graft and homologous allograft bone transplantation, with an internal fixation by locking the compression plate. This was followed by a reconstruction of the anterior and posterior cruciate ligaments and the lateral and medial collateral ligaments. There was no evidence of local recurrence at five years post-surgery.

## Introduction

Desmoplastic fibroma (collagenous fibroma) is a distinctive, rare, benign, slow-growing fibroblastic soft-tissue tumor that was first described by Evans in 1995 (1–3). The gross characteristics of the tumor are well circumscribed, with spindle to stellate fibroblasts dispersed in a fibromyxoid or densely fibrous matrix, with low mitotic activity. The condition was renamed ‘collagenous fibroma’ a year later by Nielson *et al*(4). Nielson replaced the term ‘desmoplastic fibroma’ with ‘collagenous fibroma’, since desmoplastic fibroma is misleading and suggests that the lesion consists of immature tumor cells inducing a desmoplastic response in host tissues. This opinion was consistent with that of Hasegawa *et al*(5).

At present, desmoplastic fibroma of the bone is considered the intraosseous counterpart of common soft-tissue desmoid tumors or fibromatoses (2,6). Desmoplastic fibroma of the bone has a male predominance, occurring two and a half times more often in males than in females, in individuals between the ages of 50 and 60 years old (7). Wide local resection is the recommended treatment. Written informed consent was obtained from the patient.

## Case report

**Figure 1:**
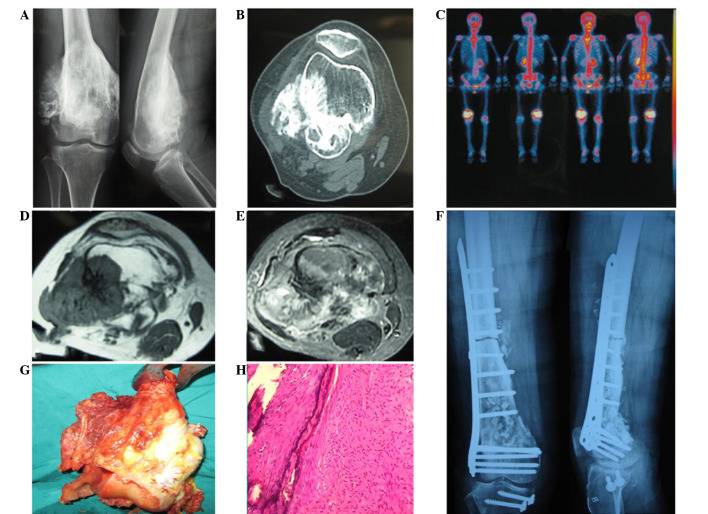
(A) X-ray showing a high-density lesion of 10 cm in diameter in the medullary cavity and around the cortical bone of the inferior femur. (B) CT revealed an inhomogeneous soft-tissue mass in the posterolateral and deep layer of the hamstrings in the right inferior femur. Calcifications were apparent as multiple small flecks; however, there was no definite boundary of the calcifications. (C) ECT showing an abnormal radioactive distribution as a mass in the right inferior femur. (D) T1-weighted MRI images revealing an irregularly-shaped expanding lesion in the right inferior femur. The lesion had a low signal intensity, similiar to the muscle tissue. (E) T2-weighted MRI images revealing an inhomogeneous hybrid signal area, including a small area of high signal intensity in the are of low signal intensity. (F) The resected bone was reimplanted using devitalized tumor bone*,* self-ilium and homologous allograft bone transplantation, with an internal fixation by locking the compression plate. This was followed by a reconstruction of the anterior and posterior cruciate ligaments and the lateral and medial collateral ligaments. (G) Gross appearance of the excised specimen showing a circumscribed mass involving the majority of the bone and the adjacent soft tissues of the thigh. (H) Microscopic examination revealing spindle cell and collagen fiber proliferation and collagen fibers intermixed with spindle cells, with low mitotic activity and no necrosis in the lesion (hematoxylin and eosin staining; magnification, ×80). CT, computed tomography; ECT, emission-CT.

A 66-year-old female presented to The Affiliated Tumor Hospital of Zhengzhou University (Henan, China) with a firm, immobile, painless and slow-growing mass of the right medial thigh that had been apparent for seven years. One week prior to treatment, the mass became intermittently painful and the movement of the right knee joint was limited. A physical examination revealed a 10-cm firm, immobile and tender mass in the right medial thigh. The temperature and color of the local skin of the mass were normal. The right knee joint was restricted to a 90° flexion, but the muscle strength and sensation of the lower limbs was normal. X-rays revealed a high-density lesion of 10 cm in diameter in the medullary cavity and around the cortical bone of the inferior femur (Fig. 1A). Computed tomography (CT) revealed an inhomogeneous soft-tissue mass in the posterolateral and deep layer of the hamstrings in the right inferior femur. Calcifications were apparent as multiple small flecks; however, there was no definite boundary of the calcifications. The lesion invaded the medullary cavity and the cortical bone (Fig. 1B). Emission-CT (ECT) revealed an abnormal radioactive distribution as a mass in the right inferior femur (Fig. 1C). MRI revealed an irregularly-shaped, expanding lesion in the right inferior femur. The lesion had a low signal intensity on T1-weighted images, which was similar to the muscle tissue, and an inhomogeneous hybrid signal area on T2-weighted images, including a small area of high signal intensity in the area of low signal intensity (Fig. 1D and E). Bone cortex dissolution and a periosteal reaction due to new bone formation were detected locally. A 92×99×96-mm mass was identified in the posterolateral section of the lesion. The mass adhered to the biceps femoris and was adjacent to the popliteal artery and vein, but no invasion to the knee joint cavity was observed. Following an open biopsy, the mass was confirmed to be a desmoplastic fibroma. The patient underwent a resection of the tumor and the accompanying bone, which was then reimplanted using devitalization *in vivo*, auto-ilium and homologous allograft bone transplantation, with an internal fixation by locking the compression plate. This was followed by a reconstruction of the anterior and posterior cruciate ligaments and the lateral and medial collateral ligaments under general anesthesia (Fig. 1F). Gross examination of the excised specimen revealed a 92×99×96-mm circumscribed mass involving the majority of the bone and the adjacent soft tissues of the thigh (Fig. 1G). A microscopic examination revealed spindle cell and collagen fiber proliferation and collagen fibers intermixed with spindle cells, with low mitotic activity and no necrosis in the lesion (Fig. 1H).

## Discussion

In 1958, Jaffe (8) first described desmoplastic fibroma of the bone as a distinct entity and a kind of osseous fibrous tumor that was previously unclassified and histologically similar to abdominal desmoid tumors. Desmoplastic fibroma of the bone is now considered the intraosseous counterpart of common soft-tissue desmoid tumors or fibromatoses (6), with a reported incidence of 0.11–0.13% among primary bone tumors (9). Desmoplastic fibroma is a rare, lytic, locally aggressive, but non-metastatic benign tumor. Almost any bone may be affected, but desmoplastic fibroma most often involves the mandible (22%), femur (15%), pelvic bones (13%), radius (12%) and tibia (9%) (9). Certain studies have emphasized that the lesion is osteolytic and does not contain a significant mineralized matrix, with a favored metaphyseal origin (10). The metaphysis and diametaphysis are equally involved, and exclusive diaphyseal involvement of a tubular bone has been reported as the rarest site of occurrence (6).

Radiographically, a desmoplastic fibroma is a lytic tumor. In the long bones, the longest dimension is aligned with the long axis of the host bone and markedly expands the shaft. Desmoplastic fibroma usually arises in the metaphysis and may reach the end of the host bone. The tumor occasionally arises in the diaphysis and an intraosseous well-defined radiolucent lesion expansion of the bone, involving the whole circumference or only part of it, is observed. When the cortex is breached, a soft-tissue mass may either invade or displace the adjacent muscles. Distinct periosteal new bone is rare, with the exception of those cases that are associated with pathological fractures (10). The lesion arising from the center of the bone has an expanded appearance without any surrounding periosteal reaction (11). The zone of transition between the tumor and the normal bone is typically narrow and well defined, but not sclerotic (12).

In a previous study of desmoplastic fibroma, cross-sectional imaging revealed a soft-tissue mass in 41% of cases on CT and in 57% of cases on MRI. The morphological appearance on the images was that of a relatively slow-growing lesion with focal aggressive features (6). CT illustrates the extent of the bone destruction and MRI visualizes the medullary and soft tissue extent of the tumor (13). CT and MRI are complementary imaging techniques in cases of suspected desmoplastic fibroma. MRI is particularly useful and is the preferred imaging modality at present to delineate desmoplastic fibroma. On MRI, the majority of soft-tissue masses have a high signal intensity on T2-weighted images, but in desmoplastic fibroma, T1-weighted images of the mass exhibit a low signal intensity, while T2-weighted images of the mass show scattered high-signal areas within a zone of low signal intensity.

The correlation between MRI and the histological findings has been described on T2-weighted images; areas of low signal intensity correspond to abundant collagen fibers and areas of high signal intensity showing marked enhancement on contrast T1-images correspond to those areas histologically consisting of fibroblasts and loose collagen fibers (14). On T1-weighted images, low signal areas represent areas with low cellularity and abundant collagen fibers (14).

Desmoplastic fibroma should be diagnosed from other soft-tissue masses with a low signal intensity on T2-weighted images, including neurofibroma, cicatricial fibroma, malignant fibrous histiocytoma, aggressive fibromatosis and calcified masses. In the absence of calcification, abundant collagen and marked hypocellularity in a soft-tissue tumor result in a decreased signal intensity on the T2-weighted pulse sequence (15).

The radiographical appearance of desmoplastic fibroma is similar to other lytic lesions. The differential diagnosis includes giant cell tumors, aneurysmal and solitary bone cysts, hemangioma, fibrous dysplasia, non-ossifying fibroma and chondromyxoid fibroma. A differentiation should also be made from primary malignant lesions, including adamantinoma, fibrosarcoma or metastatic carcinoma.

Histologically, desmoplastic fibroma is similar to a soft-tissue desmoid tumor. In the present study, a microscopic examination revealed hypocellular, bland, spindle cell proliferation, with low mitotic activity and no necrosis. These cells were associated with a large amount of intercellular collagen fibers in bundles. The cells and collagen fibers were arranged in a parallel fashion and in bundles. The lesion exhibited an infiltrative, destructive pattern with permeation of the bone marrow spaces, Haversian canals and surrounding soft tissues. Microscopic infiltrations of the tumor were present beyond the perceived macroscopic margin.

Evans (3) suggested that the most significant differential diagnostic consideration was that of a desmoid tumor, as it may have similar cytological features and is often locally aggressive. Alberghini *et al*(16) reported that desmoplastic fibroma is a myofibroblastic lesion, ultrastructurally demonstrating the presence of fibronexus junctions. Immunohistochemical studies reveal prominent myofibroblastic differentiation, which typically presents on the cytoplasmic membranes of the cells, while a desmoid tumor is fibroblastic. This ultrastructural finding is significant in the differential diagnosis between a desmoplastic fibroma and a desmoid tumor.

Treatment of desmoplastic fibroma of the bone includes curettage and intralesional, marginal or wide resection with or without replacement by allograft, cryosurgery and amputation in certain cases (17). Böhm *et al*(9) studied the recurrence rate following different methods for the treatment of desmoplastic fibroma. The recurrence rate (55%) was high in patients who underwent curettage. By contrast, the recurrence rate (17%) following the resections was much lower. In 11 of the patients who underwent wide resections with a minimal follow-up of three years (mean 6.1 years), no recurrences were reported. Therefore, wide resection is the ideal treatment for desmoplastic fibroma (12).

Bertoni *et al*(18) reported two cases, one in the scapula and one in the calcaneus, which were treated by thorough curettage (intralesional excision). The cavity in the calcaneus was subsequently filled with autogenous cortical grafts. The other four cases were treated by a wide segmental resection. Of these four tumors, the one located in the proximal fibula was treated by a resection only. The tumor in the distal femur required an endoprosthesis, but subsequently the limb was amputated due to infection. The tumor of the mid-shaft of the humerus was treated with a plate and an autogenous cortical graft. The tumor in the distal fibula was treated with an autogenous cortical graft. In no case was local recurrence observed during the follow-up. There are no studies of local recurrence or metastases at present (19,20), with the longest follow-up time recorded at 12 years. Therefore, the recommended treatment of collagenous fibroma is local surgical excision to minimize potential morbidity.
